# Methods of Delivering Mechanical Stimuli to Organ-on-a-Chip

**DOI:** 10.3390/mi10100700

**Published:** 2019-10-14

**Authors:** Kattika Kaarj, Jeong-Yeol Yoon

**Affiliations:** Department of Biosystems Engineering, The University of Arizona, Tucson, AZ 85721, USA; kkaarj@email.arizona.edu

**Keywords:** organ-on-a-chip (OOC), microfluidic device, mechanical cue, shear flow, compression, stretch, strain, syringe pump, integrated pump, passive delivery

## Abstract

Recent advances in integrating microengineering and tissue engineering have enabled the creation of promising microengineered physiological models, known as organ-on-a-chip (OOC), for experimental medicine and pharmaceutical research. OOCs have been used to recapitulate the physiologically critical features of specific human tissues and organs and their interactions. Application of chemical and mechanical stimuli is critical for tissue development and behavior, and they were also applied to OOC systems. Mechanical stimuli applied to tissues and organs are quite complex in vivo, which have not adequately recapitulated in OOCs. Due to the recent advancement of microengineering, more complicated and physiologically relevant mechanical stimuli are being introduced to OOC systems, and this is the right time to assess the published literature on this topic, especially focusing on the technical details of device design and equipment used. We first discuss the different types of mechanical stimuli applied to OOC systems: shear flow, compression, and stretch/strain. This is followed by the examples of mechanical stimuli-incorporated OOC systems. Finally, we discuss the potential OOC systems where various types of mechanical stimuli can be applied to a single OOC device, as a better, physiologically relevant recapitulation model, towards studying and evaluating experimental medicine, human disease modeling, drug development, and toxicology.

## 1. Introduction

Organ-on-a-chip (OOC) has enabled new opportunities in cell biology research through reproducing key aspects of an in vivo cellular microenvironment. One of these parameters is mechanical force, which imparts strain on cells and tissues. Such mechanical force and subsequent strain are integral parts of the cellular microenvironment that modulates the proliferation, migration, phenotype, and/or differentiation of cells. There have been extensive studies describing the cellular mechanisms where mechanical forces are transduced into biochemical signals (called cellular mechanotransduction) [1,2,3]. These responses affect the function of multicellular systems, which is critical in tissue- and organ-level health and disease. Mechanical forces provide cues for morphogenesis during organ development [4], tissue homeostasis [5], and wound healing [6], to name a few. Disease processes of fibrosis and cancer metastasis are also linked to the abnormal mechanical properties applied to tissues [7,8]. Recent studies on cellular mechanotransduction suggest that mechanical modulation of the cellular microenvironment can be used to improve the wound healing response [9], either by promoting faster wound closure or by decreasing fibrosis [10]. Generally, it is crucial to characterize the influence of mechanical forces in order to clearly understand in vivo physiology.

Mechanotransduction operates through numerous pathways that are often complex and unpredictable. OOCs can be used to recreate the in vivo biomechanical environment for studying and evaluating such mechanotransduction. In this review, we describe the latest approaches and methods used to generate mechanical stimuli on OOC systems and how they can be used towards diverse applications. Specifically, technical details of OOC system designs and equipment used are summarized and classified into several different categories. We also discuss on the potential advancement in combining different types of mechanical stimuli that can be delivered to a single OOC system.

## 2. Types of Mechanical Stimuli Utilized in Current Organ-on-a-Chip (OOC)

**Figure 1:**
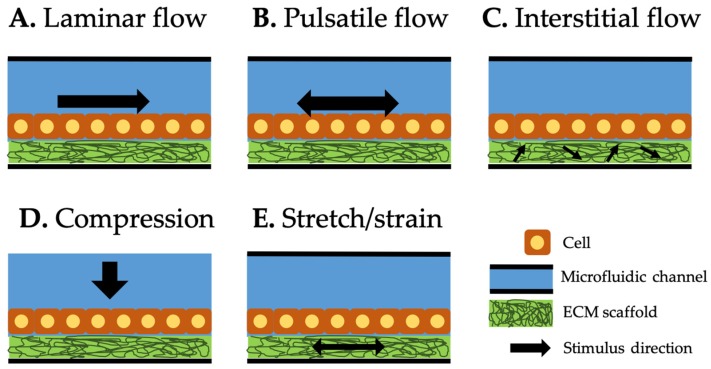
Types of mechanical stimuli integrated in organ-on-a-chip (OOC) systems. Black arrow represents the direction of each stimulus. Laminar (**A**) and pulsatile (**B**) flows along the microfluidic channel and generates laminar and pulsatile shear stresses on the cell surfaces. Interstitial flow (**C**) through the extracellular matrix (ECM) scaffold generates the shear stress to the cells attached to it. Compression (**D**) generates compressive force on top of the cells. Stretch/strain applied to the ECM scaffold (**E**) generates the force to the cells attached to it.

In vivo cells and tissues experience various mechanical stimuli, which have been recognized as key determinants of differentiated cellular functions. For example, fluid shear stress presented in the kidney induces the morphological polarization and allows epithelium to perform the transportation function, including the transport of water and sodium in response to hormonal stimulation. Mechanical stimuli commonly applied in OOC systems can be classified into three categories: shear flow, compression, and stretch/strain (Figure 1).

Liquid flow within microchannels of OOC systems usually induce the shear stress on the cells or tissues cultured in the system, thus called shear flow. Flow-induced shear stress has initially been used to study the effects of this stress on cell adhesion, mechanics, morphology, and growth in early microfluidic cell culture systems. Recent studies are focused on reproducing physiologically relevant shear stresses to understand their effects in the specific tissues and organs. Shear forces can be generated by laminar, pulsatile, or interstitial flow.

### 2.1. Laminar Flow

**Figure 2:**
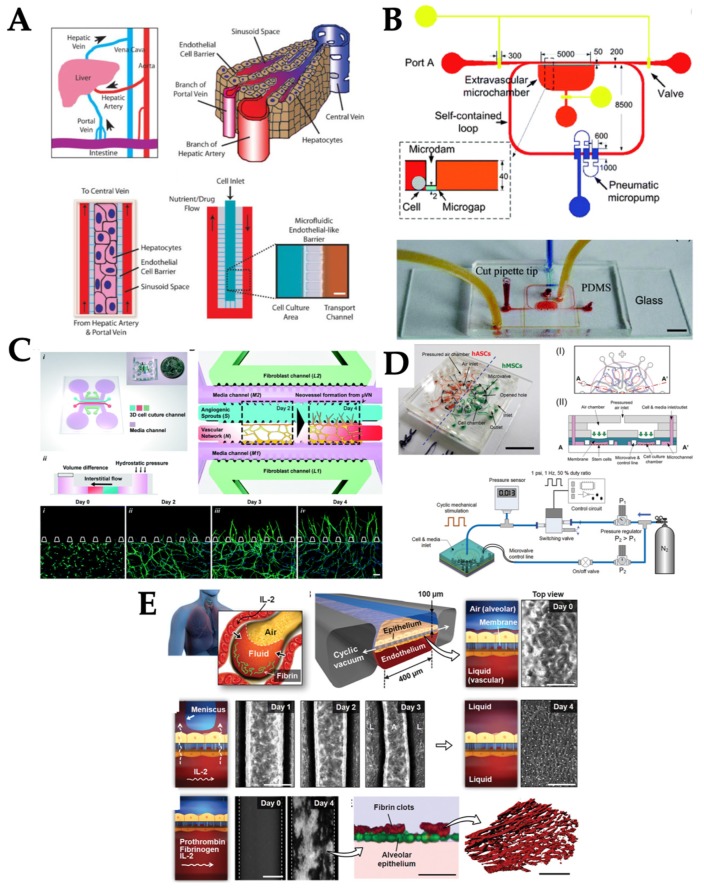
Different types of mechanical stimuli used in OOC systems. (**A**) Laminar flow presented in liver-on-chip, reproduced with permission from Reference [16]. (**B**) Pulsatile flow presented in vessel-on-chip, reproduced with permission from Reference [20]. (**C**) Interstitial flow presented in angiogenesis-on-chip, reproduced with permission from Reference [26]. (**D**) Compression stress presented in bone-on-chip, reproduced with permission from Reference [31]. (**E**) Stretch/strain presented in lung-on-chip, reproduced with permission from Reference [37].

Laminar flow is dominant in tissues and organs and subsequently OOC systems, as the dimensions are in micrometer scale (Figure 1A). This leads to a very small Reynolds number (= Dvρ/μ, where, D is tube diameter, v is flow velocity, ρ is fluid density, and μ is fluid viscosity) [11]. Such flow is characterized as laminar flow, which is a series of parallel straight paths of flow lines with parabolic profile and no mixing among them. As such, laminar flow has commonly been used in various OOC systems (Figure 2A). For example, one kidney-on-chip device reproduced luminal fluid shear stress of 0.2–20 dyn/cm^2^ (which is ~10% of the shear stress experienced by endothelial cells) in the collecting duct system of the kidney, to study the role of fluid shear stress of 1 dyn/cm^2^ in the reorganization of actin cytoskeleton [12] and translocation of water transport proteins (aquaporin-2) of inner medullary collecting duct (IMCD) cells of the kidney [13]. The kidney-on-chip device developed by Jang et al. was composed of two polydimethylsiloxane (PDMS) layers separated by a thin porous polyester membrane. The top layer was covered by IMCD cells to mimic the inside tubular system and the bottom layer was left empty for fluid flow to mimic the outside tubular system. Various environmental factors and their combinations, including 1–12 h of exposure time, 0.2–5 dyn/cm^2^ of shear stress, different concentrations of hormones, and transepithelial osmotic gradient conditions across a porous membrane, were investigated to generate various dynamic conditions for kidney tubular cells. Fluid shear stress, together with the presence of hormone and osmotic gradient, triggered the F-actin polymerization and depolymerization in both apical and basal regions of the cells, and the process is reversible. These kidney-on-chip models can be used towards studying renal physiology and pathophysiology.

Laminar flow was also used for an OOC system to study how fluid forces modulate angiogenesis (formation of new blood vessel) [14,15]. The OOC system for an angiogenesis study invented by Vickerman et al. was comprised of two parallel microfluidic channels, lined with human umbilical vein endothelial cells (HUVECs), and a central microchannel of a three-dimensional (3D) collagen extracellular matrix (ECM) that separated the two parallel microfluidic channels, into which HUVECs could migrate [14]. Similarly, Zheng et al. fabricated a microvessel network of HUVECs within collagen matrix and the 0.1 dyn/cm^2^ shear stress was delivered to the cells [15]. These OOC systems recreated the physiological environment of vascular endothelial cells for assessing angiogenesis. Laminar flow used in this system generated the tangential shear stress on the HUVECs and induced cell sprouting into the central channel. 

Another example was a liver-on-chip device, where the hepatocytes were 3D-cultured within a microfluidic channel to study drug toxicity in vitro [16]. The device was composed of highly fluidic-resistant endothelial barrier channels, which was connected to the flow channel and the cell loading channel. Media was pumped as laminar flow at the steady rate of 10–20 nL/min to mimic the mass transport properties of the hepatic microcirculation. The endothelial barrier was introduced to minimize the damage to hepatocytes caused by high shear stress. The 3D morphology of hepatocytes and liver metabolically specific functions were maintained under this continuous, laminar flow. Hepatotoxicity of the anti-inflammatory drug, diclofenac, was also investigated using this liver-on-chip model to assess the short-term (<4 h) and long-term (24 h) exposures to the liver cells and compared with the reported drug-induced injury to liver.

### 2.2. Pulsatile Flow

When the heart beats, blood is pumped to vessels throughout the body due to cycles of contraction and relaxation of the ventricles and atria, which resulted in a pulsatile flow within the transportation part of cardiovascular system (Figure 1B) [17]. Pulsatile flow has commonly been utilized in blood vessel-on-chip systems to simulate the actual pulsatile blood flow in human circulation [18]. For example, a hemodynamic microfluidic system with endothelial cells was developed mimicking the physiological pulsatile nature of the vascular system, to investigate the effect of biological factors in the blood from diabetic patients [18]. This microfluidic chip consisted of three rows for triplicate experiments to be performed at the same time. Each row has three different microchannel designs that mimic different blood vessels shapes: 600 µm-wide channel to mimic typical human arteries, 600 µm-wide at the beginning and gradually narrowing down to 300 µm, and a half-circle block with a radius of 300 µm in the middle of the 600-µm channel to mimic the blood vessel with an atherosclerotic plaque. All three channels were 150 µm deep. Pulsatile shear stress was applied at the average flow rate of 1.41 µL/s (corresponding to the average shear stress of 15 dyn/cm^2^ in 600 µm-wide channels) and at a pulse rate of 70 beats per min (bpm), both of which corresponded to the physiological conditions of typical arteries in a healthy human body. The other profile is a pulsatile flow at the elevated average flow rate of 2.22 µL/s (corresponding to the average shear stress of 23.6 dyn/cm^2^ in 600 µm-wide channels) with the elevated pulse rate of 110 bpm, to simulate the blood flow in diabetic patients. The rate of endothelial cell apoptosis increased by a factor of 1.4–2.3 with increasing glucose concentration and shear stress.

Similarly, an OOC system mimicking a microvascular transportation connecting multiple organ-on-chip systems was designed and constructed to study the long-term homeostasis of human blood vasculature and its transport function [17]. Such a multi-organ-on-chip (MOC) device consisted of two separate microvascular circuits and the insert compartments were filled with organ-equivalent culture. Pulsatile flow was delivered to the MOC at the periodically varied rates from 7 to 70 µL/min with the frequency of 144 bpm. The measured average shear stress was 25 dyn/cm^2^ in the microvascular circuit, which is within the physiological shear stress of 10–40 dyn/cm^2^ [19].

An integrated microfluidic chip cultured with endothelial cells was developed to study the endothelial cell’s morphology, cytoskeleton, and barrier formation under the self-contained loop of induced pulsatile shear stress at a physiologically-relevant level (Figure 2B) [20]. The chip is composed of three layers: a bottom layer for microgap, a middle layer for microchannels and microchamber, and a top layer for reshaping the microchannel. The average pulsatile flow rate of 0.34 nL/s was delivered to the systems, which correlated to the instantaneous shear stress from +8.15 to −5.92 dyn/cm^2^. Several in vivo physiological structures and processes were similarly represented in this chip, such as endothelium barrier, albumin transport, soluble factors diffusion, and pulsatile and oscillatory shear stress exposed to endothelial cells. These processes were utilized towards comprehensive understanding of the endothelial cell’s behavior and functions.

### 2.3. Interstitial Flow

Interstitial flow is fluid flow through or around a 3D ECM, where interstitial cells such as fibroblasts, tumor cells, tissue immune cells, and adipocytes can be found. It differs from open-channel flow, such as blood flow within vessels, in several ways (Figure 1C). For example, it generally flows at a much slower velocity because of the high flow resistance of the ECM, it moves around the cell-matrix interface in all directions rather than only on the apical side, and it can have important effects on pericellular protein gradients, particularly those that are matrix binding [21]. Microfluidic systems have been considered as a promising platform to investigate the effects of interstitial flow on cell motility. For example, a microfluidic device with two regions for ECM gel was proposed to study the effect of interstitial flow on cancer cells’ migration (metastasis) [22]. In this work, breast cancer cells were used as a cancer model and the use of two ECM gels allowed maximum flexibility in adjusting flow rates. Interstitial flow of 10 µm/s increased the number of circulating cancer cells (CTCs) as well as their velocity, but the directionality of such movement varied between cell types. Effects of interstitial flow on cancer cell invasion were also examined in order to provide a better understanding of how cancer cells gain access to the lymphatic system and manipulate their environment [23,24,25]. Bonvin et al. designed a multichamber radial flow system consisting of nine identical gel-filled chambers sharing inlet and outlet medium reservoirs [23]. Interstitial flow rate of 1–2 µm/s successfully induced the migration of cancer cells into the fibroblast-containing gel region. Similarly, Munson et al. utilized the radial flow chamber to investigate the effect of interstitial flow (average velocity of 0.7 µm/s) on the glioma (brain cancer) invasion as well as flow-induced cellular polarization [24]. A co-culture model with fibroblast and cancer cell was used to study the effect of interstitial flow (~0.5 µm/s) on cancer cell invasion [25]. In this work, interstitial flow enhanced the fibroblast migration through increased transforming growth factor β1 (TGF-β1) activation and collagen degradation, through which fibroblasts eventually reorganized collagen fibers and in turn, enhanced cancer cell invasion.

Interstitial flow also affects angiogenesis (formation of new blood vessels) as well as lymphanogenesis (formation of new lymphatic vessels). These processes are essential to both normal development and pathological processes that are heavily influenced by the dynamic mechanical environment, for example, laminar and interstitial flows experienced by endothelial cells. Effects of interstitial flow on angiogenesis and lymphanogenesis were investigated on OOC models [26,27]. Kim et al. utilized the microfluidic device composed of 6 microchannels: two peripheral media channels, two outermost channels for 3D culture of fibroblasts (secreting angiogenic biochemical cues), one central channel for a fibrin-matrix embedding endothelial cells, and one central channel for an acellular fibrin-matrix for angiogenic sprouting (Figure 2C). In the presence of interstitial flow (0.1–4 µm/s), vasculogenic organization of the microvascular network was significantly facilitated regardless of flow direction, whereas angiogenic sprouting was promoted only when the directions of flow and sprouting were opposite from each other [26]. A similar chip design was used to study the lymphanogenesis in response to interstitial flow (~1 µm/s). The growth of vessel sprouting was significantly augmented in the direction opposite the flow, while sprouting was suppressed along with the flow direction. These results imply that interstitial flow is a key mechanical regulator of tissue vascularization including the rate of morphogenesis and sprout formation [27].

### 2.4. Compression

Various types of cells experience compression stresses that are applied on top of the cells (Figure 1D). A good example is skin tissue, where the top two layers are epidermis (top, exposed layer) and dermis (just underneath the epidermis). From a physical standpoint, the main function of the dermis is to provide mechanical integrity to the skin. In normal non-injured skin, the majority of compressive and tensile forces imparted onto the skin are borne by the ECM network in the skin dermis, with little force directly applied onto the resident fibroblasts or other structures [28]. The hypodermis, innermost layer of the skin and lining between dermis and skeletal muscle, mainly consists of subcutaneous fat and serves as a shock absorber.

Another good example is cardiac tissue. It displays macroscopic contraction with the entire tissue being compressed at each contraction without collapse of the internal vasculature [29]. Microfluidic devices can be used to mimic the organ functionality through constructing multi-cellular architecture, interfacing the tissues and physiochemical microenvironments along with perfusion of the body. Several different OOCs can fluidically be connected together, mimicking the physiological connections between organs with integrating flow shear stress, mechanical compression, and cyclic strain or other physical forces, which can be used for analyzing drug distribution and organ-specific responses [30]. The complex mechanical microenvironment of organs can be replicated in vitro on OOCs, enhanced pressure can be applied to compress the tissues at a higher level than normal [31]. For example, adipocyte-derived stem cells and bone marrow-derived stem cells were cultured on microfluidic devices and exposed to dynamic hydraulic compression to evaluate the osteogenic abilities [31]. The uniform mechanical compression was provided to the array of cell culture chamber that were located along a concentric circle of the central air inlet on a microfluidic chip (Figure 2D). Dynamic mechanical compression of 1 and 5 psi(= 6.9 and 34 kPa) was evaluated to investigate its effect on the osteogenic differentiation abilities of different types of stem cells. Dynamic compression increased osteogenic ECM formation and integrin levels in both types of stem cells, leading to stimulus-enhanced bone differentiation. Bone marrow-on-chip was also used to study blood cell production under radiation countermeasure drugs [32]. Bone marrow chip exposed to gamma radiation resulted in a reduction of leukocyte production. Treatment of the chips with two potential therapeutics, granulocyte-colony stimulating factor (G-CSF) or bactericidal/permeability-increasing protein (BPI), induced significant increases in the number of hematopoietic stem cells and myeloid cells in the fluidic outflow.

### 2.5. Stretch/Strain

In addition to flow-induced shear stress, cells and tissues in the human body continuously experience organ-specific tensile and compressive forces during the normal operation of organs (Figure 1E). Compression from outside (skin tissue) and from blood (cardiac tissue) was already described in the previous section. Tensile force applied to the cellular microenvironment leads to stretch or strain to the tissue. One example is a lung-on-a-chip device that uniquely mimics the combined solid and fluid (surface-tension) mechanical stresses induced by the air-flow-induced cellular stretching and the propagation of an air–liquid meniscus in the alveoli of a human lung [33]. Previously reported in vitro models of ventilator-induced lung injury generated either cyclic stretching or air–liquid interface flow over the cells on non-stretching substrates [33,34,35,36]. This lung-on-a-chip device created more physiologically relevant mechanical microenvironments for alveolar epithelial cells during ventilation by simulating both fluid and solid mechanical stresses. This study showed that combined solid and fluid mechanical stresses (cyclic stretch and surface tension forces, respectively) significantly increased cell death and detachment compared to solid mechanical stress alone, supporting the clinical observations that cyclic stretch alone is insufficient to induce the level of the cell injury, as seen in ventilator-induced lung injury. Another example is a human-breathing lung-on-a-chip device that reconstituted a mechanically active microenvironment of the alveolar–capillary interface of the human lung [37]. Additionally, the lung-on-a-chip device reproduced the cyclic strain from the breathing movement in a human lung. The microfluidic device consisted of a two-layer central channel separated by a thin membrane for cell cultures and two side channels where a vacuum was applied for suction (Figure 2E). Cyclic stretching was achieved at a frequency of 0.2 Hz (sinusoidal wave form) with 10% strain by applying cyclic suction to the hollow side chambers, thus causing the mechanical stretching of the flexible membrane between the alveolar and capillary compartments. The lung-on-a-chip device was also used to create a human disease model-on-a-chip of pulmonary edema [37]. Such a disease model revealed that mechanical forces associated with physiological breathing motions increased vascular leakage that leads to pulmonary edema.

A model mimicking human intestinal microenvironments was developed to evaluate lipid transport across the lymphatic endothelial layer for future applications in screening drug candidates targeting intestinal lymphatics [38]. Incorporation of the presence of peristalsis and the subsequent mechanical stretching on the endothelial layer to this model may be the next critical step for better replicating intestinal lymphatics. As such, there is significant potential for microfluidic techniques to further elucidate the functionality of the lymphatic system, as well as other systems that depend on similar mechanical constraints.

Different types of mechanical stimuli and their application towards different OOC models are summarized in Table 1.

## 3. Current Methods of Delivering Mechanical Stimuli to Organ-on-a-Chips (OOCs)

**Figure 3:**
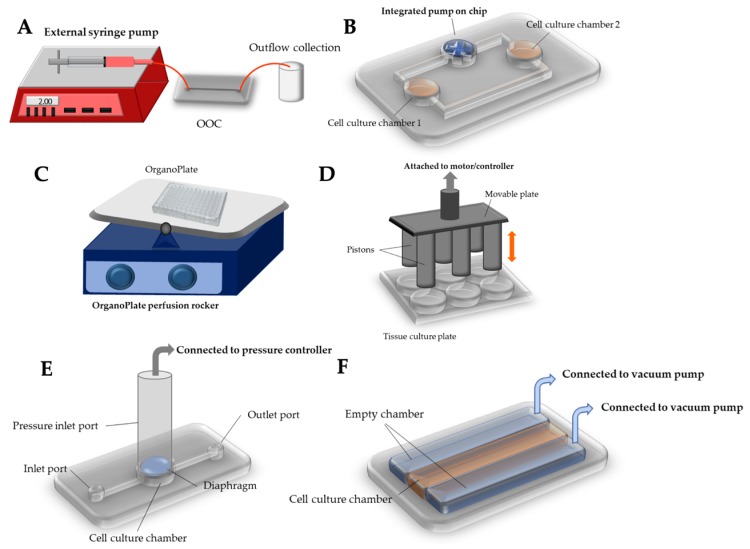
Methods and equipment used to deliver mechanical stimuli to OOC systems. (**A**) External syringe pump is delivering shear flow. (**B**) Micro-pump is integrated within a microfluidic OOC system to drive either laminar or pulsatile flow. (**C**) OrganoPlate perfusion rocker is used to generate the shear flow though the cells cultured within OrganoPlate by tilting the stage. (**D**) Compressive force is delivered to the cells on a tissue culture plate (TCP) by the pistons attached to a moveable plate that moves up and down. (**E**) Compressive force is delivered by the pressure controller to the diaphragm placed on top of the cells. (**F**) Stretch/strain is generated by periodically applying vacuum to the peripheral chambers, allowing the main culture chamber to stretch in a cyclic manner.

As described previously, mechanical stimuli found in the human body can be classified into (1) shear flow (laminar, pulsatile, and interstitial), (2) compression, and (3) stretch and strain [45], which have also been demonstrated in OOC systems. The most common stimulus used in OOC systems is shear flow as it can mimic the human body’s mechanisms to introduce medium and solution of interests to the body systems. These shear flows have been generated in diverse ways, while they can generally be classified into three categories: external pumping, internal (on-chip) pumping, and passive delivery. Figure 3 graphically illustrates various methods and equipment to deliver mechanical stimuli to OOC systems.

### 3.1. External Pumping

**Figure 4:**
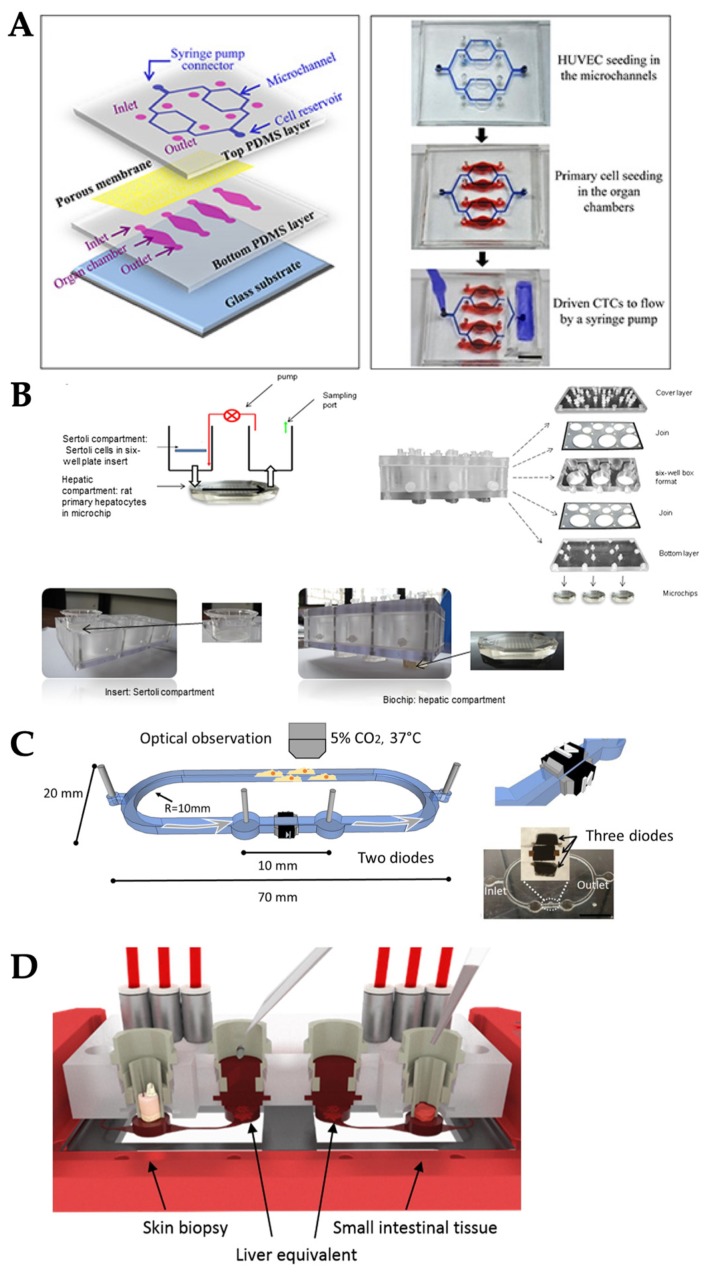
Examples of OOC systems with external and internal pumping. (**A**) External syringe pump for the OOC with endothelial cells. (**B**) External peristaltic pump for the OOC with hepatocytes and Sertoli cells. (**C**) Electro-osmosis (EOS) pumping was integrated onto the OOC with human lung cancer cells. (**D**) A peristaltic valve was integrated onto the OOC with liver-intestine and liver-skin cells. Reproduced with permission from References [49,50,51,52].

The easiest and most common method for generating shear flow in OOC is the use of an external pump, including a syringe pump or peristaltic pump (Figure 3A). A syringe pump delivers precise, accurate, and small amounts of fluid in a programmable manner, where a stepper motor pushes the plunger flange of a syringe filled with fluid, generating fluid flow to the tube connected from the end of a syringe needle. The syringe pump has popularly been used for the OOCs fabricated with polydimethylsiloxane (PDMS), which is the most frequently used substrate for many microfluidic devices as well as OOCs. For instance, cardiotoxic drug exposure was demonstrated in the PDMS-based microfluidic device cultured with human embryonic stem cell-derived cardiomyocytes with the medium flow generated and manipulated by a syringe pump [46]. Similarly, angiogenesis was demonstrated in the microfluidic device cultured with vascular endothelial cells, recapitulating in vivo blood flow along the PDMS channel [46]. Angiogenesis of vasculature tissue has also been demonstrated on an OOC system to study the angiogenic ability of different cell types, human aortic endothelial cells and HUVECs, and expression level of fibroblast growth factor under the vascular endothelial growth factor (VEGF) stimulation [47]. The syringe pump was also used to perfuse breast cancer cells (MCF7 and MDA, as circulating tumor cells) through different PDMS-based OOC systems, and the metastasis behavior of these cancer cells was observed (Figure 4A) [48]. In this work, co-culture of more than one cell type—lung-liver-bone-muscle co-culture—has been explored to provide better physiological relevance of OOC systems.

In a peristaltic pump, fluid is contained within a flexible tube fitted inside the pump casing. Its pumping principle, called peristalsis, is similar to the movements in human throat and intestines: compression and relaxation of the tube is alternated, drawing the new fluid in and propelling the existing fluid away from the pump. The peristaltic pump has also been used, albeit not as frequently as the syringe pump, to generate medium flow in OOC systems. For example, the OOC system with rat primary hepatocyte and primary Sertoli cells co-culture was demonstrated to investigate hepatic metabolism and Sertoli barrier tightness relationship (Figure 4B) [49]. The vacuum pump has occasionally been used as well: for example, in the lung-on-a-chip model, vacuum was applied to the side chambers to generate cyclic stretch (Figure 3F) [50], which is discussed in the later section. All three types of external pumps, commonly utilized for PDMS-based OOC systems, are not suitable to meet the demand of the “miniaturized” nature of OOC, due to their bulky size and requirement of an AC power source.

### 3.2. Integrated on-Chip Pumping

To overcome the size limitation of external pumping, the pumps were integrated within the microfluidic chips to miniaturize the systems (Figure 3B). For example, an electro-osmosis (EOS, voltage applied to the channel creates the bulk flow of liquid) diode pumping system was used to generate a pulsed, ultra-slow EOS flow (2.0–12.3 nL/s) through lung cancer tissue (Figure 4C) [51]. Briefly, two electro-diodes were embedded along two PDMS side walls. The electric signals were applied to the diodes directly via the electrolyte within the microfluidic channel and required no external electrical connection because the leads of the diodes physically contacted the fluid. The flow rate was adjusted by changing the electric field strength applied to the diodes from 0.5 V_pp_/cm to 10 V_pp_/cm. The diode pump-driven fluidics were characterized by intensities and frequencies of electrical inputs, pH of fluids, and fluid type. However, the long-term operation of this electro-osmotic microfluidic system required a change of fluid every hour which is not easy-to-implement for some tissue or organ models.

A miniature peristaltic pump was also integrated on the PDMS chip to generate pulsatile flow along the co-culture of liver-intestine and liver-skin tissue to investigate the efficacy of different drug dosage and administration (Figure 4D) [52]. This microfluidic chip contained the cell culture compartment, peristaltic micropump, and three PDMS membrane valves in a row. These were actuated by applying pressure. The pumping frequency and peristaltic valve pressure were modified to adjust fluid flow rate into a physiological range of a specific organ. The optimized conditions were the pump at frequency of 2 Hz, the median flow rate of 2.6 µL/min, and the median shear stress of 0.7 dyn/cm^2^. This system was stable for up to 14 days for the co-cultures of human 3D liver equivalents with human intestinal barriers.

### 3.3. Passive Delivery

**Figure 5:**
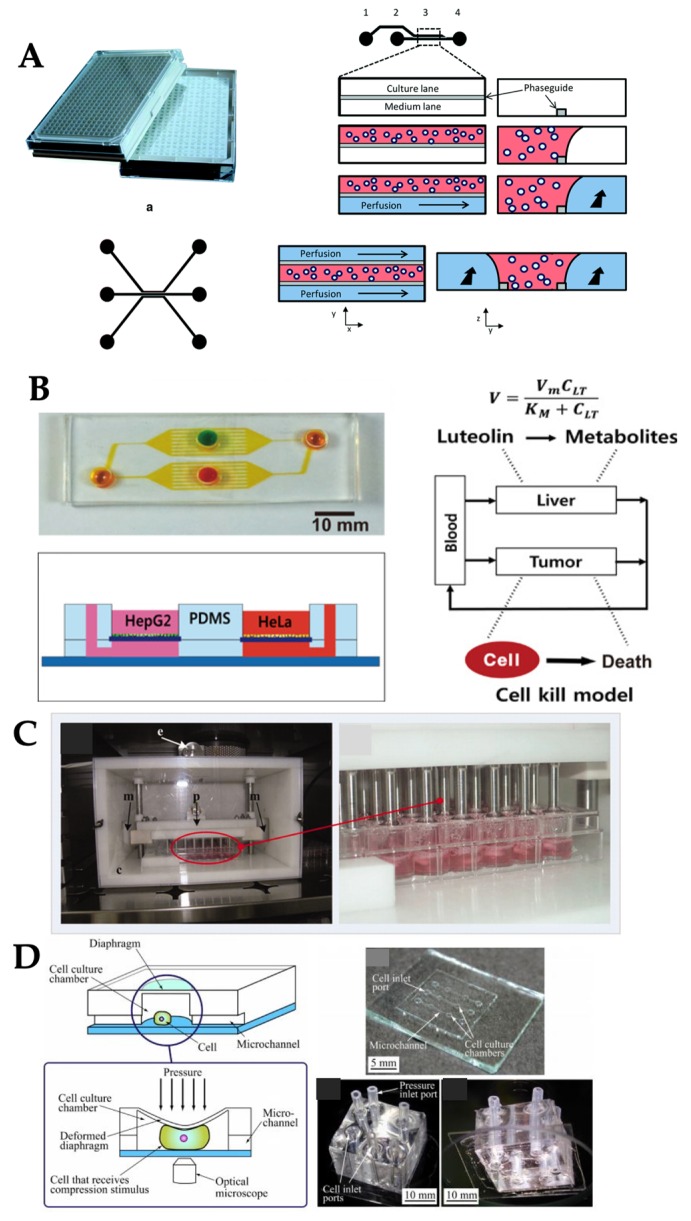
Examples of OOC systems with passive delivery and compression. (**A**) OrganoPlate was rocked to generate a passive flow through network of neurons. (**B**) Gravity flow machine generated flow to the liver and cervical cancer cells on chip. (**C**) Piston chamber generated compressive stress on the cardiac tissue within TCP. (**D**) Diaphragm and pressure controller generated compressive stress on osteoblastic cells on chip. Reproduced with permission from References [26,43,53,56].

While the internal on-chip pumping allowed for miniaturizing the OOC systems, the integration of such an internal pump onto PDMS chip is extremely complicated for both fabrication and operation. Passive control of the flow has recently emerged in the past three years as an alternative. One example is OrganoPlate, which is a commercialized microtiter plate where the cells are cultured on it and the passive flow is generated by rocking the OrganoPlate (Figure 3C). The OrganoPlate supported up to 96 tissue models on a single platform and each model usually consisted of two layers: a medium layer for passive fluid flow and an ECM gel layer. This device also offered 3D tissue culture where the cells were embedded within the ECM gel layer and also deposited as a monolayer at the inner wall of the medium layer. Continuous perfusion of medium mimicked blood flow and enabled exchange of nutrients and oxygen. This was driven by gravity levelling (difference in fluid level) using its Perfusion Rocker^TM^ and no additional pumps and tubes were necessary. OrganoPlate has been used in many OOC systems. For example, neuron tissues were cultured on OrganoPlate to investigate the stem cell differentiation (Figure 5A) [52] and co-culture of neurons-glia cell for studying neurite outgrowth [53]. While this method is simple, it still requires a moveable stage to tilt the plate for generating a passive flow. An improved method is the use of gravity-driven flow, which is also gaining popularity due to its simplicity. For example, the inlet and outlet of PDMS chip were connected to syringes containing different levels of solution. With gravity, a higher level of the medicine solution in the syringe inlet was driven to flow through liver tumor spheroids, cultured within the PDMS chip for screening the anti-cancer drug toxicity in the liver tissue [54]. Instead of syringes, the PDMS chip was placed on the computer-controlled tilting stage to drive the flow within the chip where the co-culture of liver (HepG2) and cancer (HeLa) cells existed and used to observe the pharmacokinetic and pharmacodynamic of anti-cancer drug activities (Figure 5B) [55]. Similar to the gravity-driven flow, hydrostatic pressure was used to generate interstitial flow along the vasculature tissue culture within the PDMS chip to study the angiogenesis [56]. Nonetheless, these passive flow controls can potentially result in an imprecise rate of flow and may not be practical for long-term experiments. 

### 3.4. Compression

In addition to shear stress, compression is also an important factor in certain tissues such as heart [26], bone [42], and vessel [57]. For instance, cardiac cells were cultured on alginate scaffold within a 48-well plate. These 3D cell cultures were placed underneath the compression device consisting of 48 pistons to provide the compression and medium perfusion for each culture well to study the cell behavior under mechanical stimuli (Figure 3D and Figure 5C) [42]. Compression stimuli on osteoblasts can be presented in a microfluidic device to study the real-time cellular response, for example, the changes of intracellular calcium concentration, and intracellular strain distribution after exposure to those stimuli. Compression stimuli applied to cells can be controlled by regulating the expansion of the diaphragm via pressure control (Figure 3E and Figure 5D) [58]. Cyclic compression in a sinusoidal waveform with magnitudes of 0.33, 0.5, and 1 MPa were delivered at 1 Hz frequency by a commercially available compression system (FX-5000C^TM^, Flexcell, Burlington, NC, USA), to study the differentiation of osteoblasts [59]. This commercial system regulated the positive air pressure to compress tissue samples or 3D cell cultures that were placed in between a piston and a stationary platen using their own tissue culture plate (TCP). This device allowed each piston to apply the loads up to 14 lb (=6.4 kg) into an individual well on TCP.

### 3.5. Stretch/Strain

**Figure 6:**
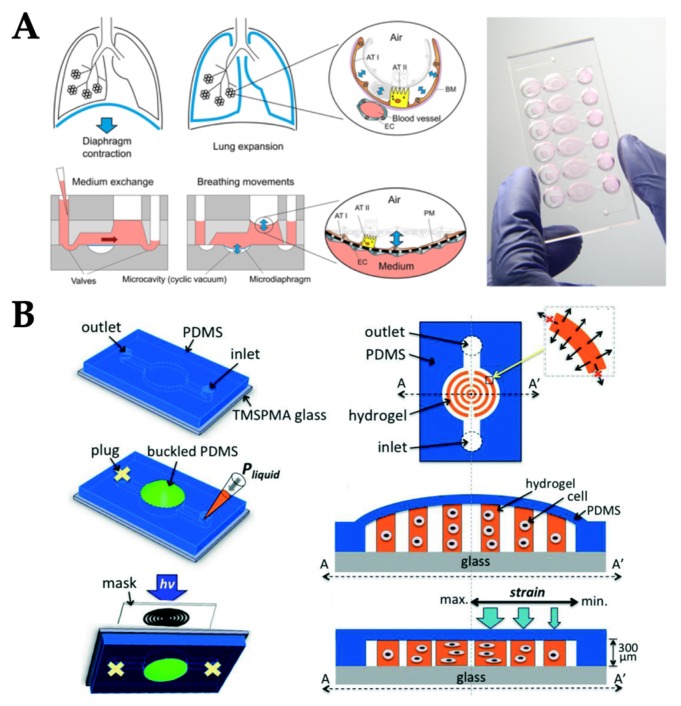
Examples of OOC systems with stretch and strain stimuli. (**A**) Lung-on-chip device was connected to the electro-pneumatic system to provide the negative pressure on the polydimethylsiloxane (PDMS) membrane (microdiaphragm), which subsequently generated the cyclic strain mimicking lung breathing mechanism. Reproduced with permission from Reference [44] (**B**) Gradient strain chip with a simple static strain delivery through the syringe at the inlet and PDMS plug at the outlet. Reproduced with permission from Reference [61].

A vacuum pump was used to generate cyclic stretchin OOCs, specifically lung-on-a-chip, as described in the external pumping section. As PDMS (the most popular material for OOC fabrication) is flexible, periodic changes in the vacuum pressure can result in stretching and/or strains to the substrate (PDMS) and subsequently to the cells attached to it. Stucki et al. fabricated a PDMS-based lung-on-chip device to study the conservation of the epithelial barrier functionality between primary human lung alveolar cells (hAEpCs) and primary lung endothelial cells for long-term co-culture and the effect of physiological cyclic strain on epithelial barrier permeability [53]. In this work, the cyclic strain was delivered to the systems by connecting a chip to an electro-pneumatic setup via access ports which was used to control the opening and closing of the valves by applying negative and positive pressures. The applied pressure deflected the 40 µm thick PDMS membrane (microdiaphragm), resulting in opening and closing the valves. The setup regulated the cyclic motion by applying a negative pressure to the access ports. The movements of the PDMS membrane were transferred to the thin, porous alveolar membrane (Figure 6A). Besides cyclic strain, the effect of gradient static-strain on cellular alignment was studied on microfluidic chip as well [60]. Fibroblasts were cultured on a concentric circular hydrogel pattern and a flexible PDMS membrane. Initially, the outlet of the flow channel on the microfluidic chip was closed with a PDMS plug. Once the liquid was injected in the flow channel, a non-continuous gradient height of hydrogel along the radius was formed. After unplugging the outlet, the PDMS membranes became flat and gradient stress was applied on the cell-encapsulated hydrogels (Figure 6B). Cell behaviors were influenced by a combination of hydrogel shape and a variety of tensile stretch guidance cues.

**Figure 7:**
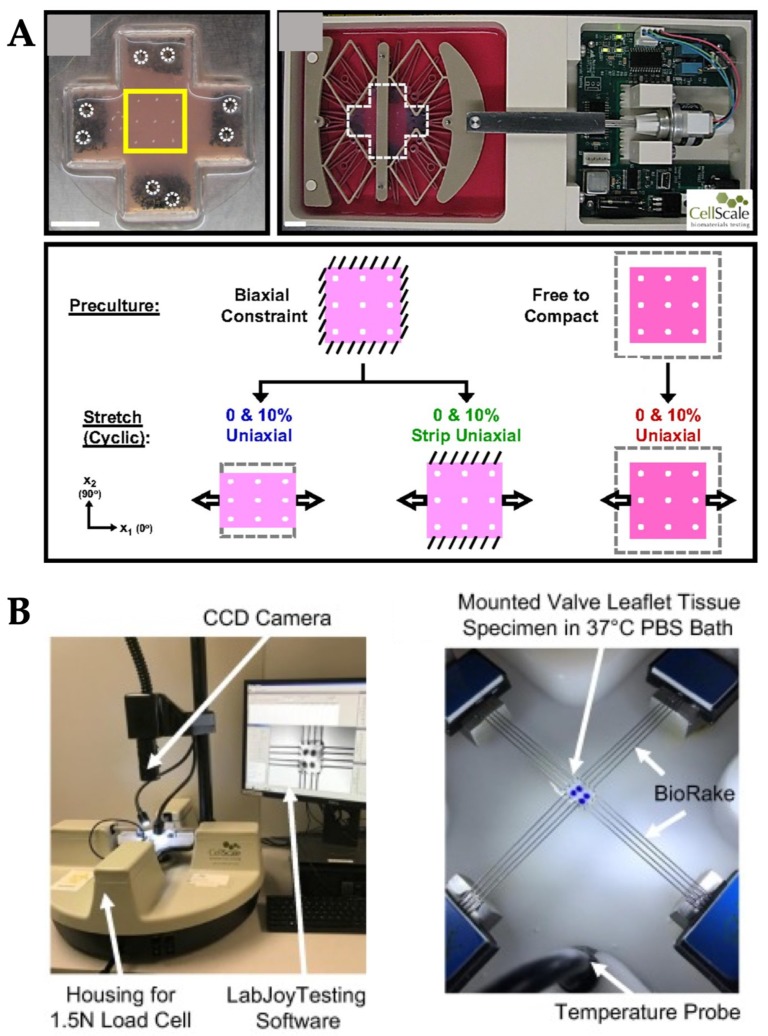
Examples of commercially available OOC systems with stretch and strain stimuli. (**A**) Microfluidic device was inserted to the MechanoCulture B1 system from CellScale Inc. to provide the biaxial mechanical stretch stimulation and evaluate its effect on cell alignment. Reproduced with permission from Reference [61]. (**B**) Porcine heart valves were positioned in the biaxial mechanical tester (BioTester, CellScale Inc.) to characterize heterogeneity of heart valve leaflet. Reproduced with permission from Reference [62].

As mechanobiological studies on OOC systems have been gaining popularity, many commercial tools have been developed that could easily deliver mechanical stimuli to the OOC systems (Figure 7). For example, the fibroblast’s ability to align in response to mechanical stimuli was tested through a series of 0% to 10% cyclic uniaxial stretch experiments in 2D and 3D cell culture by using the commercially available MechanoCulture B1 device from CellScale Inc. (Waterloo, ON, Canada) (Figure 7A) [61]. Cyclic stretch generated from commercial devices successfully induced the perpendicular fibroblast alignment in 3D culture. In addition to uniaxial mechanical stretch, biaxial stretch generated by commercialized mechanical tester (BioTester, CellScale Inc.) was used to characterize the heterogeneity and function of the heart valve leaflet (Figure 7B) [62]. The porcine atrioventricular heart valve leaflet was dissected and placed on the device where the various loading ratios and stress-relaxation tests were performed on each region of tissue sample. Briefly, membrane tension applied by the device in the circumferential and radial directions were 100 and 50 N/m for different heart valve leaflet, and varying ratios of membrane tension loading in each direction was applied with eight loading/unloading cycles to investigate all possible physiological tissue deformations. Similarly, proteomic analysis of mitral valve anterior leaflets under 10% cyclic radial strain at a frequency of 1 Hz was investigated by using the commercialized MCT6 uniaxial stretcher (CellScale Inc.), where the tissue was placed between the two clamps of the device [63]. After mechanical stretch exposure, the endothelium of the heart valve was removed to test how endothelium mediates the mitral valve’s response to stretch. This work showed a significant finding on new protein groups that might be involved in the mitral valve’s response to mechanical stretch.

## 4. Delivering Multiple Types of Mechanical Stimuli Simultaneously

A specific organ requires a specific microenvironmental stimuli to achieve an organ-level function, for example, lung cells require breathing-derived mechanical stretch [64]. The general approach for fabricating an OOC is to identify key aspects of the geometrical, mechanical and biochemical microenvironment of the tissue. With these key aspects identified, a microengineering approach is used to recapitulate them on a chip, under the researcher’s full control. Then, isolated cells are introduced on the chip and subjected to the designed stimuli.

**Figure 8:**
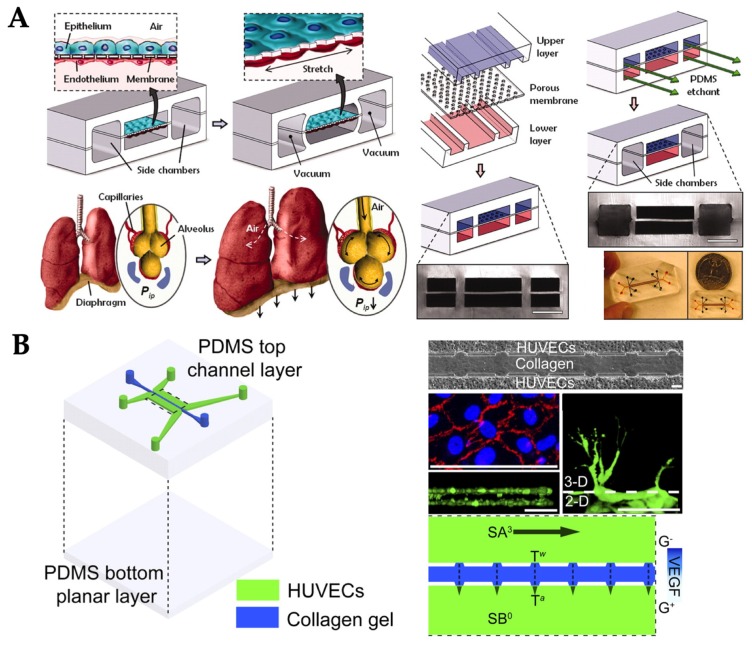
Examples of OOC systems with multiple types of mechanical stimuli. (**A**) Lung-on-chip. Reproduced with permission from Reference [64]. (**B**) Endothelial cells-on-chip. Reproduced with permission from Reference [66].

Some tissues experience multiple types of biochemical and/or mechanical stimuli. While multiple biochemical cues can be applied to a single chip relatively easily, it is not easy to do so for mechanical stimuli. For example, lung tissues are exposed to periodic mechanical force within each respiratory cycle: air flow in the air–epithelium interface, blood flow in the endothelium–blood interface, and stretching on the epithelial–endothelial interface. The lung-on-chip model has therefore been developed integrating all of these mechanical stimuli on a single device [64]. The microfabricated lung-on-a-chip consisted of compartmentalized PDMS microchannels to form an alveolar–capillary barrier on a thin, porous, flexible PDMS membrane coated with ECM. The device recreated physiological breathing movements by applying a vacuum at a frequency of 0.2 Hz to the side chambers through a vacuum pump and causing mechanical stretching (5–15% strain) of the PDMS membrane forming the alveolar–capillary barrier. Alveolar epithelial and human pulmonary microvascular endothelial cells were grown on either side of a thin porous membrane and were exerted by 20 µL/h (fluid shear stress of 0.1 dyn/cm^2^) media flow via the two channels separated by a membrane (Figure 8A). Culture medium for alveolar epithelial cells was gently aspirated from the upper channel on day five when the cells reached confluency to form an air–liquid interface. After that, culture media for both cell types were delivered to the lower channel to feed the epithelial cells on their basolateral side. This device can serve as a tool for studying lung diseases and related screening of drugs. While the model was still small and relatively simple, complex geometries were utilized that might prevent from easy reproduction and/or long-term operation of the device.

Similarly, Musah et al. developed a glomerulus-on-chip (GOC) that is analogous to the previously described lung-on-chip [65]. This GOC model efficiently facilitated the differentiation of human-induced pluripotent stem (hiPS) cells into functional human podocytes, cells that wrap around capillaries of the glomerulus and play a crucial role in selective permeability in blood filtration. It successfully recapitulated the natural tissue–tissue interface of the glomerulus and the clearance of albumin and inulin when co-cultured with human glomerular endothelial cells. The GOC can be used for drug development and specifically personalized-medicine, for example Adriamycin-induced albuminuria and podocyte injury. The fabrication and design were comparable to the lung-on-chip model developed by Huh et al. [64], where the alveolar–capillary barrier on a thin laminin-coated porous membrane was replaced with the urinary–microvascular interface, which recreated the physiology of human glomerulus in kidney, while the two vacuum channels parallel to the fluidic channel were maintained. The peristaltic pump was used to perfuse the fluidic channels at the volumetric flow rate of 60 µL/h, corresponding to the shear stress of 0.0007 dyn/cm^2^ in the 1 mm × 1 mm urinary channel and 0.017 dyn/cm^2^ in the 1 mm × 0.2 mm microvascular channel. While the cyclic stretching (10% strain) was applied to the GOC by the vacuum regulator at an amplitude of −85 kPa and frequency of 1 Hz. Adriamycin, a cancer treatment drug, was used as an example of drug toxicity studies by continuously being infused through the microvascular channel for up to five days. The toxicity of this drug was assessed by a series of protein assays.

Another example is the formation of new blood vessels or vasculature, e.g., angiogenesis. Vascular endothelial cells, or simply ECs, are continuously exposed to shear flow (blood and interstitial flows) and thus, experience shear stress tangential to the endothelial surface [66]. Shear stress causes ECs to secrete biomolecular signals [53], leading to vascular structure remodeling [67], cytoskeleton rearrangement [68,69,70], and transcriptional gene expression. Furthermore, ECs are exposed to interstitial flow, which acts as a transverse force across the vessel wall. Interstitial flow above a certain threshold promotes capillary formation [14,71]. In one OOC model, both tangential shear stress from luminal flow and stress from interstitial flow were applied to the ECs on a chip to study angiogenesis [66]. Briefly, this OOC consisted of two peripheral microfluidic channels for HUVECs cultured on fibronectin coated surface and central channel for collagen/fibronectin gel (Figure 7B). A syringe pump was used to deliver a laminar flow with the shear stress of 3 dyn/cm^2^, the physiological level of shear stress in veins or venules, and the interstitial flow within the collagen/fibronectin channel was measured as 0.0045–0.064 dyn/cm^2^. The results showed that fluid shear stress enhanced the EC sprouting and interstitial flow promoted morphogenesis and sprout formation. 

Finally, osteogenesis, the differentiation of mesenchymal stem cells (MSCs), is another excellent example that can be heavily influenced by multiple types of mechanical stimuli: compression, shear stress, strain, stretch, and hydraulic forces [72]. Virtually all types of mechanical stimuli should be represented in an osteogenesis model, making the OOC design and fabrication extremely challenging. As such, OOC demonstration of osteogenesis is still ongoing.

## 5. Mechanobiological Studies and Real-time Microscopic Imaging

Mechanical stimuli on OOC systems can also be utilized to improve mechanobiological studies, i.e., how an individual cell responds to the applied mechanical stimuli. To this end, cells and cellular responses must be monitored in real-time without destroying the cells within the OOC systems. For example, a customized miniature incubator was designed and fabricated to allow the real-time microscopic observation of cultured cells in the aforementioned EOS pumping systems, where the microscope lens was positioned above the microfluidic chip and the miniature incubator (Figure 4C). This culturing platform with live-cell imaging was effectively used for assessing cellular biophysical changes under fluid conditions [51].

**Figure 9:**
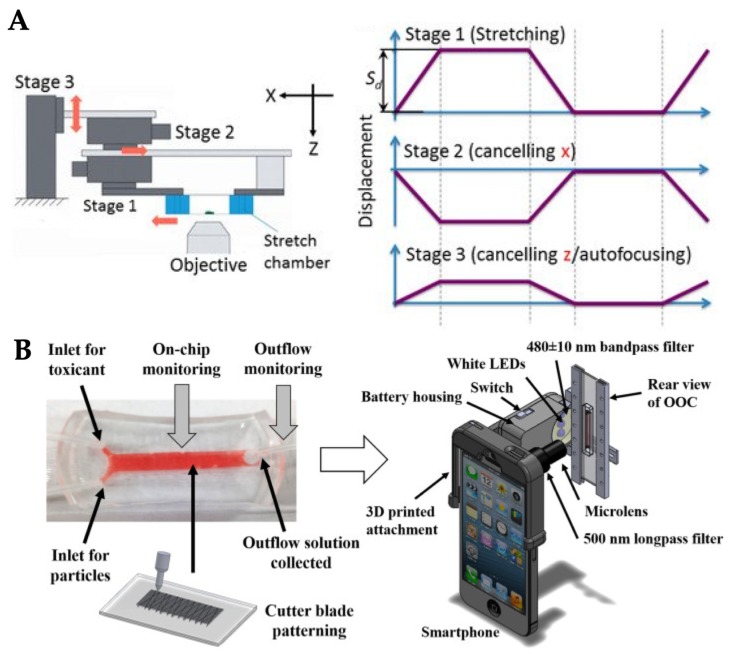
Examples of mechanobiological models and real-time monitoring devices for OOC systems. (**A**) Three motorized stages delivered cyclic stretching and a microscope imaged cellular local displacement. Reproduced with permission from Reference [73]. (**B**) Smartphone-based fluorescence microscope attached to an OOC device for in situ monitoring of nephrotoxicity. Reproduced with permission from Reference [75].

Cellular response to mechanical stretch can occur as quickly as within a few seconds and sudden disruption of cell cytoskeletons can also occur at any point during such a cyclic stretch. Huang et al. developed a cell stretching device and a real-time imaging system to investigate rapid cellular responses to two phases of cyclic stretch stimulation (Figure 9A) [73]. Three motorized stages were used to deliver the cyclic stretching to a cell culture chamber while the target cells were maintained in the same position. The microscope was positioned underneath the stage system to collect cell images. The cell stretching and imaging system was able to determine the maximum displacement of the target cells in the horizontal and vertical directions and to capture the sudden disruption of cell–cell junctions in response to two phases of cyclic stretch at the frame rate of 30 fps (frames per second). This time resolution was greatly improved from the other cellular mechanobiological studies. Similarly, a commercial microscope-mountable uniaxial stretching system (STB-150W, Strex Inc., San Diego, CA, USA) was used to study the real-time single cell morphological deformation in response to mechanical stretch (Figure 9B) [74]. This commercial device stretches the cell culture chamber from both sides to enable the cells to remain inside the viewing area of a microscope. Nevertheless, the aforementioned systems still required bulky microscope apparatus to image the cells. To resolve this problem, a smartphone-based fluorescence microscope was developed and attached to an OOC device to construct a small and handheld OOC system (Figure 9C) [75]. A microscope attachment was modified with an additional LED light source and bandpass filters and the smartphone captured and processed fluorescent images. This smartphone-based OOC system was used to study nephrotoxicity of drugs with reduced assay time and production cost, while improving assay specificity and ease-of-use.

## 6. Conclusions

The emerging OOC technologies have been widely utilized toward physiologically relevant models for understanding human physiology, studying diseases, drug development, and toxicology. In OOCs, dynamic microenvironments around cells, tissues or organs have been recapitulated within microfluidic chips. Physiologically relevant mechanical stimuli are one of the crucial microenvironments that affect the cellular responses and functions. In order to deliver mechanical stimuli to OOC systems, external syringe pumps have most frequently been used to drive the liquid through the microfluidic channel with high precision and in a programmed manner. Pumps were also integrated within the microfluidic chip to minimize the size. Simpler delivery methods have also been demonstrated, including rocking, passive delivery, or applying hydrostatic pressure. Pistons and pressure controllers on the diaphragm were used to apply the compression stress to OOC systems. Delivery of more than one type of mechanical stimuli has also been suggested and demonstrated to better recapitulate the physiologically relevant microenvironment for tissue and organ, for example, lung-on-a-chip and kidney-on-chip, that is exposed to shear flow as well as cyclic strain. Successful fabrication and operation of OOC can enable us to closely mimic specific physiologically relevant microenvironments, and to provide an accessible, inexpensive, and readily manipulatable platform for rapid and definitive testing of both fundamental and applied biological hypotheses.

## Figures and Tables

**Table 1:** Summary of different types of mechanical stimuli and delivering mechanisms towards various organ/tissue models and their applications.

Mechanical Stimuli	Delivery Method	Organ/Tissue Model	Applications	Reference
Laminar flow	Passive delivery (gravity-driven)	Liver	Improvement and maintenance of cell viability under drug exposure	[16]
	Pressure regulator	Liver	Real-time monitoring of metabolic function of liver and drug-induced mitochondrial dysfunction	[39]
	Syringe pump	Kidney	Transportation, absorption, toxicity, and pathophysiology of kidney	[40]
Pulsatile flow	Peristaltic on-chip micropump	Blood vessel	Mimicking blood circulation systems connecting two different organs-on-chip towards long-term homeostasis	[17]
	Syringe pump	Blood vessel (endothelial cells)	Endothelial cell’s integrity and apoptosis in the blood of diabetic patients	[18]
	Pneumatic pump	Blood vessel (endothelial cells)	Barrier formation and permeability of endothelial cells	[20]
Interstitial flow	Peristaltic pump	Breast cancer	Cancer cell’s invasion in response to interstitial flow	[22]
	Passive delivery (hydrostatic pressure-driven)	Brain cancer (glioblastoma stem cells)	Patient-derived glioblastoma stem cell’s invasion in response to interstitial flow	[41]
	Passive delivery (hydrostatic pressure-driven)	Blood vessel (endothelial cells)	Angiogenesis: vasculogenic formation and angiogenic remodeling in response to interstitial flow and vascular morphogens	[26]
Compression	Pressure regulator	Bone (stem cells)	Osteogenic ability of adipose tissue- and human bone marrow-derived stem cells in response to hydraulic compression	[31]
	Compression device	Heart	Formation and functions of cardiac muscle tissue during the stage of compression	[42]
	Pressure controller	Bone (osteoblasts)	Real-time monitoring of single cell response to compressive stimuli	[43]
Stretch/strain	Vacuum and syringe pump	Lung	Visualization and characterization of inflammatory processes in response to bacteria at the alveolar–capillary interface	[37]
	Pneumatic pump	Lung	Conservation of the epithelial barrier property between human lung alveolar cells and primary lung endothelial cells under long-term co-culture and cyclic strain	[44]
	Syringe pump	Connective tissue (fibroblasts)	Effect of static strain on cellular alignment of fibroblasts encapsulated in hydrogel	[45]
